# Short and Intense Tailor-Made Notched Music Training against Tinnitus: The Tinnitus Frequency Matters

**DOI:** 10.1371/journal.pone.0024685

**Published:** 2011-09-15

**Authors:** Henning Teismann, Hidehiko Okamoto, Christo Pantev

**Affiliations:** 1 Institute for Biomagnetism and Biosignalanalysis, University of Muenster, Muenster, Germany; 2 Department of Integrative Physiology, National Institute for Physiological Sciences, Okazaki, Japan; Virginia Commonwealth University Medical Center, United States of America

## Abstract

Tinnitus is one of the most common diseases in industrialized countries. Here, we developed and evaluated a short-term (5 subsequent days) and intensive (6 hours/day) tailor-made notched music training (TMNMT) for patients suffering from chronic, tonal tinnitus. We evaluated (i) the TMNMT efficacy in terms of behavioral and magnetoencephalographic outcome measures for two matched patient groups with either low (≤8 kHz, N = 10) or high (>8 kHz, N = 10) tinnitus frequencies, and the (ii) persistency of the TMNMT effects over the course of a four weeks post-training phase. The results indicated that the short-term intensive TMNMT took effect in patients with tinnitus frequencies ≤8 kHz: subjective tinnitus loudness, tinnitus-related distress, and tinnitus-related auditory cortex evoked activity were significantly reduced after TMNMT completion. However, in the patients with tinnitus frequencies >8 kHz, significant changes were not observed. Interpreted in their entirety, the results also indicated that the induced changes in auditory cortex evoked neuronal activity and tinnitus loudness were not persistent, encouraging the application of the TMNMT as a longer-term training. The findings are essential in guiding the intended transfer of this neuro-scientific treatment approach into routine clinical practice.

## Introduction

Chronic tinnitus is a disease that deserves attention and study, because to this date there is no standard cure. Chronic tinnitus is one of the most common auditory disorders, currently affecting 10 to 15% of the adult general population [1]. Unfortunately, patients often fail to cope with or compensate their tinnitus, and then their quality of life can be considerably limited. Many patients even exhibit severe co-morbid disorders like insomnia or depression [2].

Tinnitus is likely a result of maladaptive plasticity in the central auditory pathway [3]. The original tinnitus signal is most often triggered by hearing loss. Based on auditory neural input deprivation, the excitation-inhibition balance in the central auditory pathway is disturbed, most probably by the weakening of inhibitory networks. Consequently, maladaptive brain changes lead to neuronal hyperactivity, increased neuronal synchrony, and possibly burst firing. All these neuronal phenomena have been shown to be associated with the tinnitus perception [4].

In order to effectively cure tinnitus, the neurons that underlie this auditory phantom perception need to be identified and targeted. It has been argued that the target neurons are those coding frequencies affected by hearing loss, for instance because tinnitus spectra and the spectra of the most effective tinnitus maskers resemble frequency regions affected by hearing loss [5], [6]. However, even though most tinnitus patients indeed have hearing loss as detectable by a standard audiometric examination, there also are tinnitus patients who have normal standard hearing thresholds [7], or patients with hearing loss in whom there is no clear relationship between tinnitus pitch and audiogram profile [8]. Furthermore, many people with hearing loss do not have tinnitus.

An essential supplement to measuring the hearing threshold is the determination of the perceived tinnitus pitch. In patients with *tonal* tinnitus, usually the “tinnitus frequency” (i.e. the frequency that sounds most similar to the tinnitus [9]) can be matched, and it has been demonstrated that auditory cortex neurons coding the tinnitus frequency are involved into tinnitus perception [10], [11], [12]. Thus, these neurons are a potential treatment target. However, it should be noted that the reliable determination of the tinnitus frequency is not at all trivial: a high-frequency audiometer covering the frequency range up to 16 kHz [13] should be utilized, pitfalls like octave confusions need to be considered, and the reliability of matching increases when patients are trained [14].

As mentioned before, there is no standard cure for tinnitus [4]. One major problem is that there are several different treatment target candidates in the brain (e.g. auditory cortex, thalamus, dorsal/ventral cochlear nuclei, inferior colliculus, cochlear nerve, or the limbic system [15]). Another problem is to hit potential targets with the necessary precision (e.g. using tools like transcranial magnetic stimulation, or transcranial direct current stimulation [16]). However, it appears plausible to assume that the auditory cortex would principally be a treatment target, because the tinnitus percept arises here, and changes in auditory cortex must exist when tinnitus is present [17].

The seemingly most obvious avenue to target tinnitus is via the auditory modality, using for instance broadband noise to mask and habituate the tinnitus perception [18]. However, auditory stimulation treatments might often be too unspecific, i.e. they do not take into account parameters of the individual patient profile, such as the tinnitus sound quality, the tinnitus frequency, or the hearing threshold.

In a previous study [12], [19], assuming that maladaptive plastic changes generally are reversible [20], [21], [22], we developed and evaluated a customized auditory stimulation treatment strategy (tailor-made notched music training (TMNMT)), which individually targets auditory cortical areas coding the tinnitus frequency. We succeeded to reverse maladaptive plasticity processes associated with the tinnitus perception to a certain degree, probably by reducing the excitability of auditory neurons that coded the tinnitus frequency, resulting in subjective tinnitus loudness reduction. Similar findings were also reported by [13].

However, our previous study raised several critical questions. The answers to these questions would have implications for the application of this neuro-scientific treatment approach during routine clinical practice. For instance, an important query is whether the TMNMT effects remain persistent over time after training cessation. Another relevant issue concerns patient profile variables that may influence TMNMT efficacy. Eventually, it remains to be investigated how long the TMNMT has to last until effects become measurable and noticeable.

In the present study, employing behavioral and magnetoencephalographic (MEG) outcome measures, we investigated (i) the efficacy of a short and more intensive variant of the TMNMT (i.e. 24 hours of notched music distributed over 5 subsequent days), (ii) the durability of the induced TMNMT effects (by employing a follow-up observation phase of 31 days), and crucially, (iii) the relevancy of the tinnitus frequency for TMNMT efficacy in two groups of matched tinnitus patients with chronic tonal tinnitus and either low (i.e. ≤8 kHz) or high (i.e. >8 kHz) tinnitus frequencies.

## Results

The two patient groups that were compared in terms of TMNMT efficacy (tinnitus frequency ≤8 kHz (N = 10) vs. tinnitus frequency >8 kHz (N = 10)) did not significantly differ in age (t(18) = −0.57, p = 0.58), tinnitus duration (t(18) = −0.27, p = 0.79), general psychopathological distress (as assessed with the SCL-90-R inventory [23]) (t(18) = −1.4, p = 0.162), and hearing loss (there was neither a significant main effect of group (F(1,18) = 0.1, p = 0.76), nor were there significant interactions of group with ear (F(1,18) = 0.21, p = 0.65), frequency (F(12,216) = 1.13, p = 0.34), or ear *and* frequency (F(12,216) = 0.52, p = 0.90)). Furthermore, before TMNMT onset (i.e. at baseline) tinnitus-related distress (as assessed with the Tinnitus Questionnaire [24]) (t(18) = 0.63, p = 0.54) and tinnitus loudness diary values (t(18) = 1.35, p = 0.19) did not significantly differ between groups (Table 1). Therefore, the two groups were comparable regarding both relevant tinnitus-related characteristics as well as baseline values of the dependent variables. Retrospectively, neither total music listening times (t(18) = 1.07, p = 0.299) nor subjective music enjoyment (t(18) = −0.28, p = 0.785) did significantly differ between the two patient groups.

**Table 1:** Patient characteristics and baseline values of outcome measures broken by patient group.

Patient groups	Patient characteristics	Values [mean ± sd]
Tinnitus frequency ≤8 kHz	Age [years]	32.2±8.2
	Tinnitus duration [years]	5.1±6.4
	Tinnitus-related distress [0 – 40 points]	8.5±6.8
	General psychopathological distress [0 – 90 points]	21.7±13.7
	Subjective tinnitus loudness [0 – 100 points]	61.2±11.8
Tinnitus frequency >8 kHz	Age [years]	34.4±9.1
	Tinnitus duration [years]	5.8±4.9
	Tinnitus-related distress [0 – 40 points]	6.7±5.9
	General psychopathological distress [0 – 90 points]	33.0±30.0
	Subjective tinnitus loudness [0 – 100 points]	51.7±18.8

To assess effects of the TMNMT on tinnitus perception and tinnitus-related evoked auditory cortex activity, as well as to study the persistency of such potential effects, we normalized the values of the dependent variables obtained at the points in time **(i)** shortly after TMNMT completion, **(ii)** 3 days after TMNMT completion, **(iii)** 17 days after TMNMT completion, and **(iv)** 31 days after TMNMT completion relative to the baseline values (formula: (values at **(i)**, **(ii)**, **(iii)**, and **(iv)**/values at baseline)-1) separately for the two patient groups, and tested whether the normalized values at the different points in time were significantly different from zero (if so, there would be a significant change relative to baseline) by means of planned comparisons. To account for multiple comparisons, we controlled the false discovery rate at 5 % [25]. t-values and corresponding p-values are summarized in Table 2.

**Table 2:** Statistical t- (df = 9) and (unilateral) p-values of the calculated planned comparisons broken by patient group and as functions of outcome measure and time point.

Patient groups	Outcome measures	Shortly after TMNMT^a^	3 days after TMNMT	17 days after TMNMT	31 days after TMNMT
Tinnitus frequency ≤8 kHz	Tinntus-related distress [t (p)]	−1.99 (0.0385)	−1.52 (0.065)	−2.11 (0.0175)*	−2.38 (0.0085)*
	Tinnitus loudness [t (p)]	−2.3 (0.0235)*	−0.96 (0.1805)	−2.15 (0.016)*	−1.12 (0.132)
	N1m [t (p)]	−0.34 (0.372)	−2.14 (0.0165)*	−1.97 (0.0245)*	n.m.^1^
	ASSR [t (p)]	−0.03 (0.488)	0.43 (0.332)	0.39 (0.349)	n.m.
Tinnitus frequency >8 kHz	Tinntus−related distress [t (p)]	−0.06 (0.4775)	0.48 (0.3225)	0.47 (0.317)	0.99 (0.161)
	Tinnitus loudness [t (p)]	−0.69 (0.2535)	0.2 (0.42)	1.12 (0.1325)	−0.67 (0.252)
	N1m	n.a.^2^	n.a.	n.a.	n.a.
	ASSR	n.a.	n.a.	n.a.	n.a.

^a^ Tailor-made notched music training.

*Significant; false discovery rate controlled at 5 %. ^1^ Not measured. ^2^ Not analyzable.

**Figure 1:**
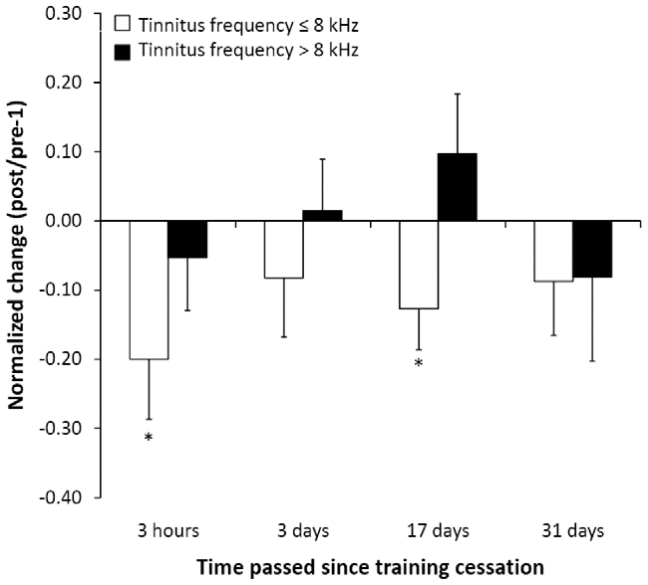
Tinnitus loudness ratios. Normalized tinnitus loudness changes relative to baseline at four time points after training completion for both patient groups. White bars represent the low tinnitus frequency (≤8 kHz) group, black bars represent the high tinnitus frequency (>8 kHz) group. Asterisks denote significant changes, the error bars denote standard errors of the mean. Positive values indicate aggravation, and negative values indicate alleviation.

**Figure 2:**
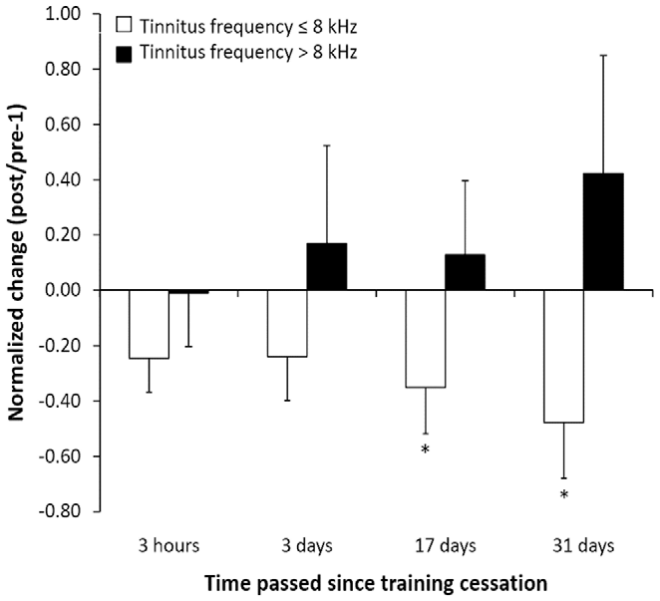
Tinnitus-related distress ratios. Normalized tinnitus-related distress changes relative to baseline at four time points after training completion for both patient groups (arrangement according to Figure 1).

**Figure 3:**
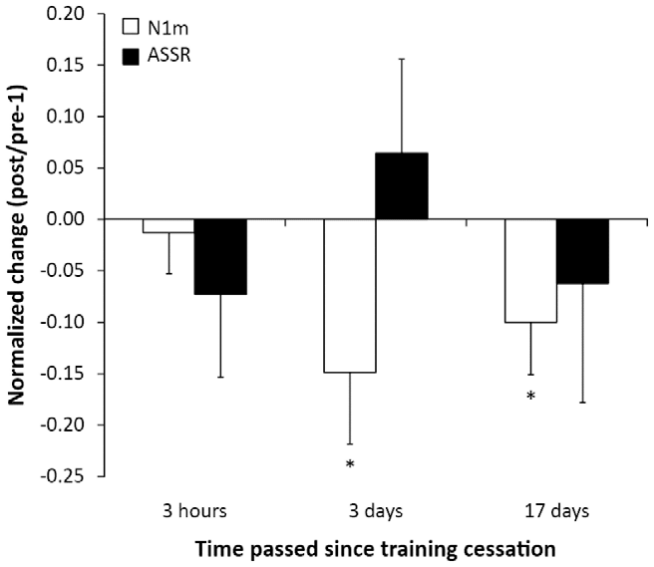
N1m and ASSR source strength ratios. Normalized N1m and auditory steady-state response (ASSR) changes relative to baseline at three time points after training completion for the patient group characterized by tinnitus frequencies ≤8 kHz. White bars represent N1m source strength, black bars represent ASSR source strength. Asterisks denote significant changes, the error bars denote standard errors of the mean. Positive values indicate increment, and negative values indicate decrement. Please note that for the patient group characterized by tinnitus frequencies >8 kHz auditory evoked fields are not available due to technical limitations of the MEG sound delivery system (limit  = 8 kHz).

As shown in Figure 1, for the patients with tinnitus frequencies ≤8 kHz, **(i)** shortly after TMNMT completion normalized tinnitus loudness was significantly reduced (t = −2.3, p<0.03). There were no significant changes in tinnitus-related distress (Figure 2), normalized N1m ratio, and normalized auditory steady-state response (ASSR) ratio (Figure 3). Moreover, there was no significant difference in normalized loudness diary values before vs. after TMNMT units (t(9) = 0.58, p = 0.29). **(ii)** 3 days after TMNMT completion, there was a significant reduction in normalized N1m ratio (t = −2.14, p<.02) (Figure 3). There were no significant changes in normalized tinnitus loudness, normalized tinnitus-related distress, and normalized ASSR ratio (Figures 1, 2, and 3). **(iii)** 17 days after TMNMT completion, normalized tinnitus-related distress (t = −2.11, p<0.02) (Figure 2), normalized tinnitus loudness (t = −2.15, p<0.02) (Figure 1), and normalized N1m ratio (t = −1.97, p<0.03) (Figure 3) were significantly reduced. There was no significant change in normalized ASSR ratio (Figure 3). **(iv)** 31 days after TMNMT completion, normalized tinnitus-related distress was significantly reduced (t = −2.38, p<0.01) (Figure 2). There was no significant change in normalized tinnitus loudness (Figure 1).

For the patients with tinnitus frequencies >8 kHz, there were no significant changes in normalized tinnitus loudness or normalized tinnitus-related distress at any of the four points in time (Figures 1 and 2). Due to technical limitations (see methods section), N1m and ASSR data are not available for this group.

## Discussion

For the first time, we succeeded to demonstrate that short and intensive TMNMT could effectively reduce subjective tinnitus loudness and tinnitus-related distress. Crucially, we found this effect only in patients with tinnitus frequencies ≤8 kHz. While the loudness reduction effect was already significant shortly after TMNMT completion, then fluctuated and vanished, the distress reduction was only a trend at this point in time, which then however manifested and stabilized circa two weeks after TMNMT completion. Moreover, in patients with tinnitus frequencies ≤8 kHz, there was a significant N1m source strength reduction three days after TMNMT completion, which seems to have slowly decayed, yet which still outlasted until the next MEG measurement two weeks later.

The loudness reduction effect observed here in tinnitus patients with tinnitus frequencies ≤8 kHz replicates the effect seen in our previous study [12], [19], however on a different, much shorter time scale. Thus, it seems to be possible to significantly alleviate subjective tinnitus loudness by listening to tailor-made notched music over the course of only a few days, when the daily listening time is considerable. Therefore, the TMNMT becomes potentially feasible for many tinnitus patients.

It arises the question what are the neuronal mechanisms that could underlie the observed tinnitus loudness reduction effect. We suggest that TMNMT would have induced a circumscribed auditory functional deafferentation [26] or transient sensory input deprivation, respectively. This deprivation may have rather rapidly led to a reduction of excitability of auditory cortex neurons coding the notched frequencies, among them the tinnitus frequency. The excitability reduction might have been caused by the (transient) strengthening of locally weakened inhibitory impact [27] in the auditory cortex of the patients [12]. For instance, there is evidence in adult rat barrel cortex that inhibitory synapse density could be dominantly and proportionally (relative to excitatory synapse density) increased within 24 hours of sensory stimulation [28], [29].

The neurons coding the tinnitus frequency are likely involved into tinnitus perception [11], [12], [19], [27]. However, given that the patients studied here did not exhibit severe hearing loss (and therefore vast tonotopic reorganization would not be expected), these neurons could probably still be excited via their original thalamo-cortical tuning (in this case by auditory input corresponding to the tinnitus frequency, which was used as test stimulus during the MEG measurements). At the same time, it would be possible to inhibit these neurons via their neighbors in frequency space. Thus, when the patients were listening to their notched music, due to the notch the neurons coding the tinnitus frequency would have been hardly excited. Their neighbors, however, would have been excited strongly, and they could have projected lateral or co-tuned inhibition [30] to the target neurons coding the tinnitus frequency. Over time, this type of stimulation could have led to reduction in the excitability of auditory cortex neurons coding the tinnitus frequency, and eventually to changes in tinnitus perception.

Importantly, the loudness reduction effect did not seem to be persistent: already 3 days after TMNMT completion, it was no longer measurable. We interpret this only short-lasting effect duration as indication that the induced plastic changes were merely functional and therefore transient in their nature – to elicit more stable and persistent effects, i.e. large-scale structural changes [31], the training needs to be performed over a longer period of time, presumably at least several weeks or even months. This assumption is also strongly supported by studies investigating rehabilitative training approaches for different diseases thought to be associated with maladaptive brain plasticity, for instance focal hand dystonia [20], and phantom limb pain [21], [32].

The tinnitus-related distress reduction effect observed here in tinnitus patients with frequencies ≤8 kHz exhibits a rather different time course than the loudness reduction effect. While there is merely a reduction trend directly after the TMNMT, the effect becomes significantly larger and more stable over time. At first glance, this development appears somewhat surprising. However, it should be considered that the tinnitus questionnaire measured emotional and cognitive distress. The questionnaire items target tinnitus-related cognitions, thoughts, and feelings, whose alteration may need some time to reach the conscious level. Hence, from a psychological point of view, the delayed distress reduction effect may reflect the subjects' awakening that **(i)** the TMNMT indeed had been effective (e.g. given that the tinnitus became louder again sometime after TMNMT completion), that **(ii)** the TMNMT could be repeated anytime, and that **(iii)** it could be performed over a longer period of time, potentially increasing its effectiveness.

**Figure 4:**
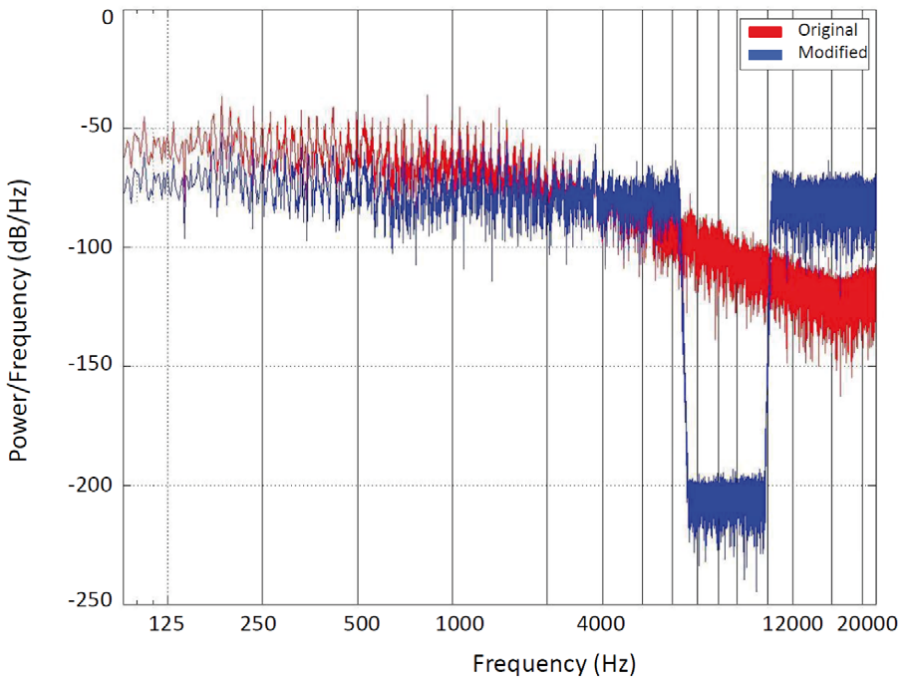
Music spectra. Exemplary frequency spectra of original (red) and modified (i.e. flattened and notched) (blue) music pieces. Here, the notch is centered at 7100 Hz.

An additional crucial finding was that the TMNMT efficacy depended on the tinnitus frequency. Even though we had relatively amplified high frequency music energy during the filtering process (Figure 4), and despite having utilized a headphone that reliably transduced very high frequencies, the TMNMT was on average only effective for patients with tinnitus frequencies ≤8 kHz, but not for patients with frequencies above this value. From a theoretical viewpoint, this finding is plausible for several reasons: **(i)** the sensitivity of the human cochlea is comparably low for very high frequencies [33]. Thus, much larger sound pressure levels must be used to make very high frequencies audible. **(ii)** Age-related hearing loss progresses from the highest to the lower frequencies [33]. Hence, this factor adds to the cochlea's general relative insensitivity for very high frequencies. **(iii)** Music usually contains relatively little very high frequency energy. **(iv)** Eventually, during listening the patients might involuntarily have paid most attention to the rather low frequencies (for instance to the voices of the singers), which are more relevant for music perception and enjoyment than the rather high frequencies. Taken together, these arguments demonstrate that it would be challenging to effectively suppress the activity of target neurons coding very high tinnitus frequencies, and it remains to be investigated whether the TMNMT could principally work for tinnitus patients with tinnitus frequencies >8 kHz. On the one hand, it appears reasonable to assume that for such cases the treatment stimulus should contain a sufficient amount of high frequency energy. On the other hand, we presume that it would be important that the treatment stimulus and strategy remained interesting or motivating enough to activate attention- and reward-related networks of the brain thought to promote plastic change. One possibility would be to further enrich the music spectrum in the high frequency range, for instance by adding high-pass noise.

The results showed a significant reduction in N1m source strength for tinnitus patients with tinnitus frequencies ≤8 kHz (Figure 3). Notably, this effect was not significant shortly after TMNMT completion, but three days later, with effect size becoming lesser 2 weeks later. Basically, this reduction effect replicates an effect seen in our previous study [12], [19]. The observed decay of the effect over the course of the two weeks following TMNMT completion suggests that the short-term training-induced changes were not persistent.

Based on our previous findings [12], [19], we presumed that tinnitus frequency-evoked N1m amplitude change and tinnitus loudness change were associated. Yet, in the present study, the N1m amplitude change did not correspond as well to the tinnitus loudness change as in the previous study, possibly because subjective tinnitus loudness does not only depend on neural activity in auditory cortex [3], [4]. Nonetheless, there is evidence that listening to notched music can reduce notch center-frequency evoked (here: tinnitus frequency-evoked) N1m amplitude on very short [26] and rather long time scales [12], [19]. Further, listening to music (or noise) that is notched around the tinnitus frequency can alleviate tinnitus loudness and annoyance [12], [13], [19]. Still, the relationship between tinnitus frequency-evoked N1m amplitude change and tinnitus loudness change (or changes in other aspects of tinnitus perception) may be rather complex. For instance, it is known that both N1m amplitude [34] and tinnitus perception [35] are sensitive to parameters such as alertness, attention focus, or mood, and the impact of these parameters on N1m amplitude and tinnitus loudness may not necessarily be equivalent. Moreover, tinnitus perception is multifaceted, and the *variability of changes between different aspects of tinnitus perception* (e.g. loudness, awareness, annoyance, or distress) is presumably higher on rather short compared to rather long time scales. Thus, it is less likely to find a simple correlation between change in tinnitus perception and change in auditory cortex neural activity on a rather short time scale. However, regarding these arguments and our previous findings [12], [19], and considering the present observation that the overall time courses of tinnitus frequency-evoked N1m amplitude change and tinnitus loudness change (i.e. reduction and return to baseline) are in line, we suggest that the reduction of neural activity in auditory cortex could be closely related to subjective tinnitus loudness alleviation.

In our previous study [12], [19], in addition to the N1m effect, we had observed a significant ASSR source strength reduction induced by the long-term TMNMT, which was positively correlated with the tinnitus loudness reduction. Yet, a significant ASSR change was not found in the present study. However, the arguments presented above regarding the N1m basically apply to the ASSR as well. Moreover, it may be that plastic changes in the primary auditory cortex (as reflected by ASSR) would need longer to develop than corresponding changes in non-primary auditory cortex (as reflected by N1m), particularly if top-down modulation is expected to play a critical role. During the present study, the patients had been instructed to listen to their training music with as much pleasure as possible, and therefore top-down modulation probably has taken place. Furthermore, while primary auditory cortex activity is most strongly modulated by bottom-up input, non-primary auditory cortex activity is strongly shaped by both bottom-up *and* top-down input [36]. Moreover, there is evidence indicating that non-primary auditory cortex may be more plastic than primary auditory cortex [37], [38]. Eventually, it has been argued [37] that attention-related modulations in primary auditory cortex may be *driven* by non-primary auditory cortex attention-related changes, given that the alterations are more robust here [38], [39].

In conclusion, this study demonstrates that it was possible to (i) transiently alleviate subjective tinnitus loudness, and to (ii) more steadily reduce perceived tinnitus-related distress in patients with chronic tonal tinnitus, not more than moderate hearing loss, and tinnitus frequencies ≤8 kHz by means of short and intensive TMNMT. Neurophysiological TMNMT effects were measurable in non-primary auditory cortical areas. The direction (i.e. reduction) and the time course (i.e. build-up and decay) of neuronal activity change induced by the training imply that the short-term TMNMT could partly and transiently reverse maladaptive plastic changes contributing to the tinnitus perception. Taken together, towards the goal of transferring the TMNMT approach into routine clinical practice, the findings motivate (i) the administration of the TMNMT as a long-term treatment, (ii) the targeted advancement of the TMNMT for patients with tinnitus frequencies >8 kHz, and (iii) the systematic utilization of attention-, emotion-, and motivation-related brain networks for the purpose of TMNMT efficacy.

## Materials and Methods

### Participants

We recruited 24 adult patients with chronic (≥3 months) tonal (i.e. peep- or whistle-like) tinnitus, and without severe hearing loss (≤50 dB HL between 125 and 16000 Hz, measured in octave steps for frequencies up to 1 kHz, and in ½ octave steps for frequencies above 1 kHz, utilizing the Orbiter 922DH clinical audiometer (GN Otometrics, Denmark)). 20 patients completed the 5 days TMNMT. 4 patients (2 patients per group) dropped out during the TMNMT due to underestimation of participation effort. The completers were divided into two groups based on their tinnitus frequencies: (1) patients with tinnitus frequencies ≤8 kHz (N = 10), and (2) patients with tinnitus frequencies >8 kHz (N = 10). The value 8 kHz was chosen in order to achieve comparability to our previous long-term TMNMT study [12], where we had included only patients with tinnitus frequencies ≤8 kHz.

In order to reduce possible placebo effects, the patients were explained before study onset that they would randomly receive one out of two treatments: either (1) the target music training, or (2) the alternative music training. In fact, all patients received the target music training (i.e. training (1)). The alternative music training (training (2)) was not administered. The patients were informed that in case of both trainings the music would be modified in an individual (and audible) way based on the tinnitus frequency. However, patients were not told how exactly the music would be modified in any of the two training versions to guarantee complete blinding. After completion of the study, the patients were debriefed. Patients gave written informed consent for the participation in the study. The study was performed in accordance with the Declaration of Helsinki. The study was approved by the Ethics Commission of the Medical Faculty, University of Muenster, Germany.

### Music modification

The patients provided 6 hours of their most enjoyable music in CD audio quality (sampling rate 44100 Hz, 16 bit, stereo). In a first processing step, the music energy spectrum was digitally “flattened” by redistributing energy from lower to very high frequency ranges. In a second processing step, the frequency band of one octave width centered at the individual tinnitus frequency was digitally removed from the music energy spectrum by means of a Butterworth notch filter (bandwidth: (tinnitus frequency/√2) to (tinnitus frequency × √2); order: 150) (Figure 4).

### Music training

The TMNMT was performed over the course of 5 subsequent days. The patients were instructed to listen to their training music for 3 hours on days 1 and 5, and for 6 hours (2 times 3 hours) on days 2, 3, and 4. Patients listened to the notched music via supplied closed headphones (Beyerdynamic DT-770, 32 Ohm Edition) and with comfortable loudness (patient-driven). Listening times had to be documented on a daily basis.

### Behavior measurements

Tinnitus-related distress was measured with the E+C subscale of the German version of the Tinnitus Questionnaire [24] (i) shortly before TMNMT onset, (ii) shortly after TMNMT completion, and (iii) 3 days, (iii) 17 days, and (iv) 31 days after TMNMT completion.

Moreover, the subjective tinnitus loudness status was measured by means of a visual analogue scale (VAS) throughout the study on a daily basis, beginning 14 days prior to TMNMT onset (familiarization phase), and ending 31 days after TMNMT completion (tinnitus loudness diary). During (i) the TMNMT, (ii) the 7 days prior to TMNMT onset, and (iii) the 9 days following TMNMT offset, subjective tinnitus loudness was measured 4 times per day at times of day that corresponded to the times before and after music listening during the training phase (e.g. at 8:00, 11:15, 14:00 and 17:15). At the remaining days, the loudness was measured once per day (always at the same time of day, e.g. always at 8:00). Subjects were instructed to perform the loudness estimation always at one and the same quiet location. Moreover, during the training phase subjects were supposed to wait for 15 minutes after finishing a music listening unit before they made the loudness measurement.

### MEG measurements

Auditory evoked fields (AEF) were measured by means of a 275 channel MEG system (Omega 275, CTF, VSM MedTech Ltd.) in a silent magnetically shielded room. However, for patients with tinnitus frequencies >8 kHz, the AEFs could not be measured with sufficient quality, which was a consequence of the spectral sound transmission properties of the tubal system utilized to deliver the sound stimuli to the patients' ears (frequencies >8 kHz are strongly attenuated). Therefore, for this group AEFs are not available. The baseline MEG measurement took place directly before training onset. Course measurements were performed (i) shortly (approx. 3 hours) after training completion, (ii) 3 days after training completion, and (iii) 17 days after training completion. To evoke auditory fields, two different sound stimuli were delivered randomly to either the left or the right ears of the patients. The carrier frequency of one stimulus corresponded to a patient's individual tinnitus frequency. The carrier frequency of the other stimulus was 500 Hz (control stimulus), which was distinctly separate from the tinnitus frequencies of all included subjects. The tinnitus frequency stimulus evoked activity from a cortical region contributing to the tinnitus perception, while the control stimulus evoked activity from a cortical area not involved in the tinnitus perception.

The stimuli had duration of 1.0 s. The initial 0.3 s were sinusoidal, whereas the remaining 0.7 s were amplitude-modulated with a modulation frequency of 40 Hz and a modulation depth of 100 %. The utilization of such stimuli allows the recording of both clean transient N1m and sustained auditory steady-state responses (ASSR) simultaneously [40]. The loudness of the control stimulus was set to 45 dB above individual hearing threshold. The tinnitus frequency stimulus was matched in loudness to the control stimulus prior to the baseline measurement. The power difference between the two test stimuli was kept identical across all course measurements. The sound onset asynchrony was randomized between 2.0 and 3.0 s.

**Figure 5:**
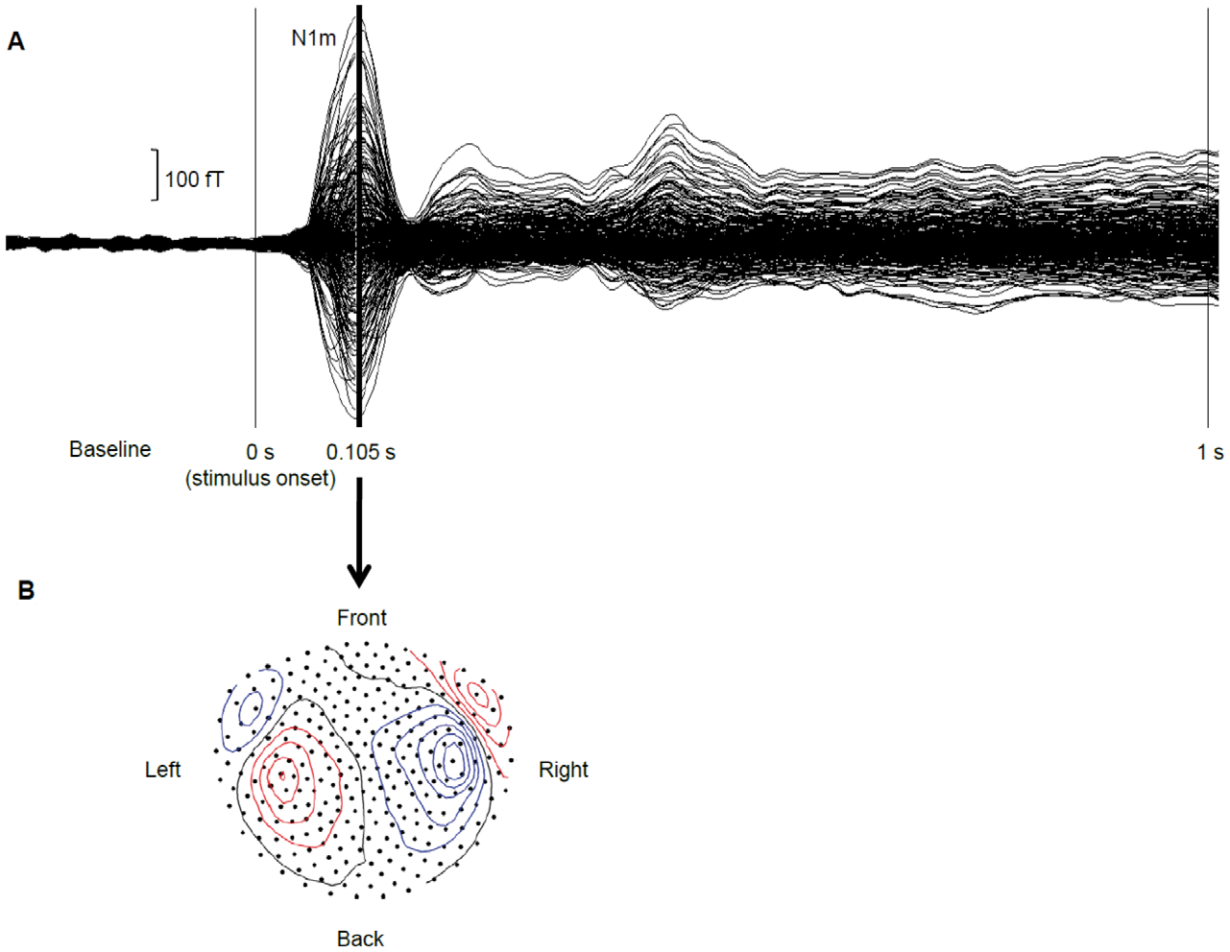
Auditory evoked field. **A** Example of a 30 Hz low-pass filtered auditory evoked field exhibiting a clear N1m response peaking 0.1 s after stimulus onset. **B** Example of a contour plot corresponding to the 0.01 s time interval prior to the N1m peak shown in A. The plot displays clear dipolar patterns over left and right hemispheres.

The contour maps of both N1m (Figure 5) and ASSR responses displayed clear dipolar patterns over both hemispheres, motivating the use of a single dipole model for source analysis. For N1m analysis, the grand-averaged magnetic fields were baseline corrected and 30 Hz low-pass filtered. The 0.01 s time window prior to the N1m peak was used for equivalent current dipole estimations (one dipole per hemisphere), and the maximal N1m source strength for each condition (tinnitus frequency vs. control frequency) and each hemisphere was calculated by using the source space projection technique [41]. For ASSR analysis, the grand-averaged magnetic fields were baseline corrected and 32 to 48 Hz band-pass filtered. The source space projection technique (based on N1m sources) was used to calculate the average ASSR source strengths across the time interval from 0.7 to 1.0 s for each condition (tinnitus frequency vs. control frequency) and each hemisphere.

In order to eliminate effects of head position differences on source strength within subjects between course measurements, we calculated ratios between the source strengths evoked by the tinnitus frequency stimulus and the source strengths evoked by the control stimulus.
